# Traumatic epidural hematoma in a patient with severe schizencephaly

**DOI:** 10.1002/ccr3.5150

**Published:** 2021-12-11

**Authors:** Van Trung Hoang, The Huan Hoang, Vichit Chansomphou, Dung Tien Doan

**Affiliations:** ^1^ Department of Radiology Thien Hanh Hospital Buon Ma Thuot Vietnam; ^2^ Department of Radiology Savannakhet Medical‐Diagnostic Center Kaysone Phomvihane Laos; ^3^ Department of Radiology Can Tho University of Medicine and Pharmacy Can Tho Vietnam

**Keywords:** central nervous system malformation, computed tomography, epidural hematoma, extradural hematoma, schizencephaly

## Abstract

Schizencephaly is a rare congenital brain structural abnormality that is not clearly understood and has no specific treatment yet. Therefore, cases related to it should be added to the literature. This report aims to introduce a rare case of severe schizencephaly co‐occurring with post‐traumatic intracranial epidural hematoma.

## Graphical abstract

Schizencephaly is a rare congenital developmental malformation, for which there is no specific treatment yet. Schizencephaly should be differentiated from lesions associated with gray matter abnormalities and cerebrospinal fluid‐containing defects.

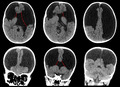

## 1 INTRODUCTION

Schizencephaly is known as a split‐brain disorder that is a rare congenital developmental malformation of the central nervous system. It is characterized by clefts in the cerebral tissue that are commonly located in the Sylvian region and can be unilateral or bilateral.^1^ They connect the pial surface to the ependymal surface of the ventricle through the white matter to the pial surface and are usually covered by gray matter with infrequent focal microgyria.^2^ We report a 5‐year‐old boy who has an incidental diagnosis of schizencephaly after traumatic brain injury.

## 2 CASE PRESENTATION

**FIGURE 1:**
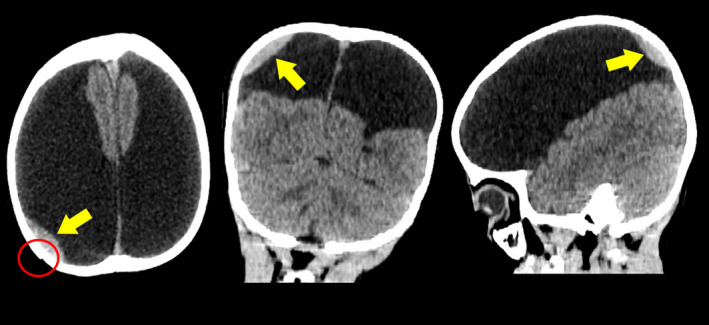
Axial, coronal, and sagittal CT images show skull bone fracture (red circle) and epidural hematoma (yellow arrows) in the right parietal region

**FIGURE 2:**
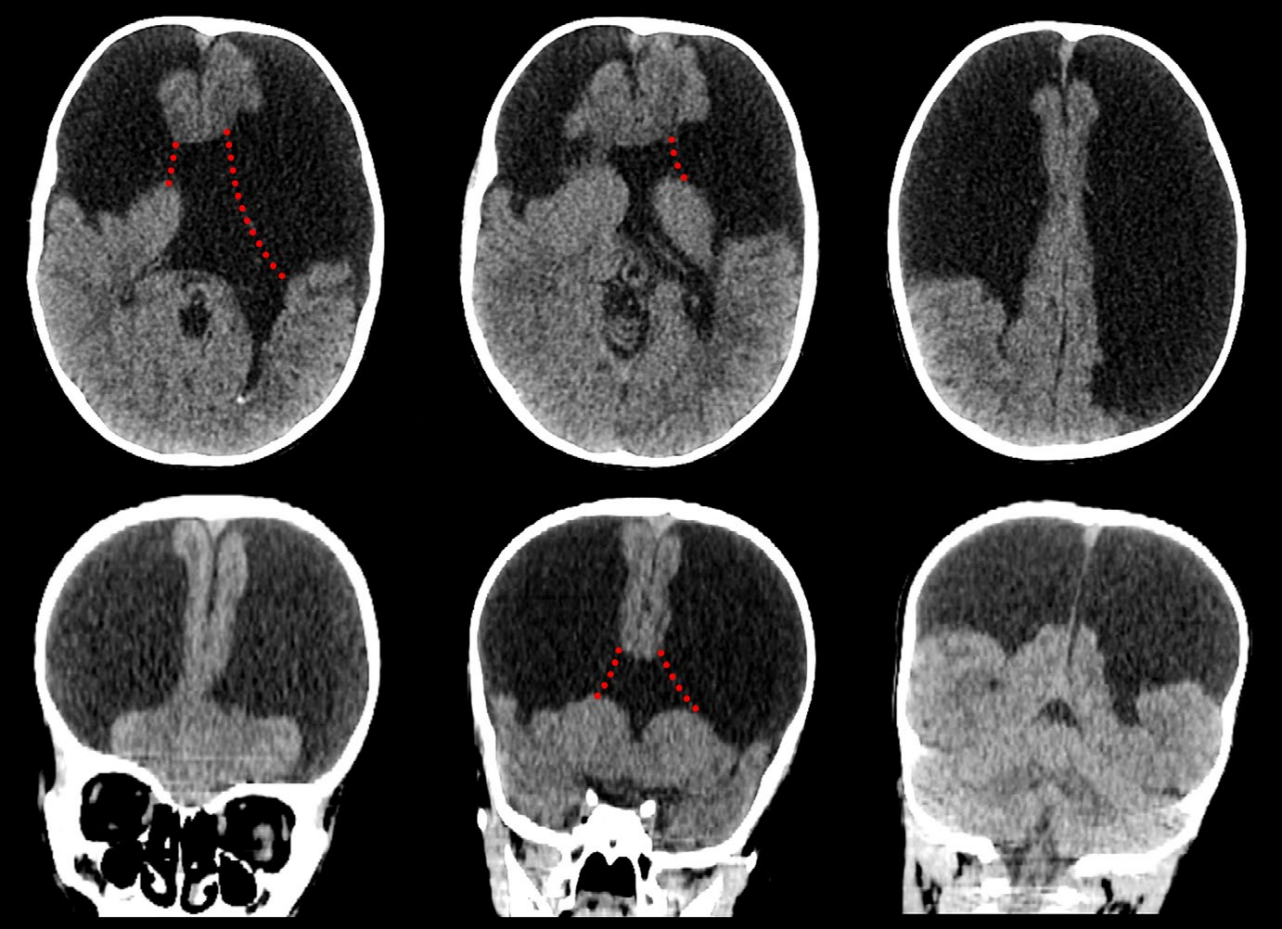
CT images show large brain defects in most of the bilateral frontal, parietal, and temporal lobes communicating with the ventricles by wide clefts (red dotted lines). The septum pellucidum and corpus callosum are absent. Note that MRI is more sensitive than CT for detecting gray matter abnormalities

A 5‐year‐old boy was admitted to the hospital in a state of traumatic brain injury due to a fall and hit his right parietal head against the wall. His history was presented with motor and mental retardation accompanied by occasional seizures. Family history was unremarkable. Vital signs including pulse, temperature, blood pressure, and respiratory rate were all normal limits. Laboratory evaluations were nothing unremarkable. Physical examination revealed mild scalp swelling in the right parietal region. The patient presented with a headache with delayed mental and motor response. He underwent a computed tomography (CT) scan of the head, which showed skull fracture with epidural hematoma in the right parietal region (Figure 1). Furthermore, there were severe brain defects with bilateral wide clefts and absence of septum pellucidum and corpus callosum (Figure 2). Due to the motion artifacts, magnetic resonance imaging technique was not performed despite the best efforts. The patient received only conservative treatment and was discharged from the hospital later. The boy was followed up showing remission of the hematoma after 2 weeks. However, the growth retardation remained unchanged as no cure for the problem was found.

## 3 DISCUSSION

Schizencephaly was first described by Wilmarth^3^ in 1887, then Yakovlev and Wadsworth^4^ proposed a two‐type classification (closed‐lip type: not communicating with the ventricular system; open‐lip type: communicating with the ventricular system) in 1946, and Griffiths^5^ proposed a three‐type classification (type 1: trans‐mantle column of abnormal gray matter but no evidence of a CSF‐containing cleft; type 2: CSF‐containing cleft present, abutting lining lips of abnormal gray matter opposed; type 3: CSF‐containing cleft present, non‐abutting lining lips of abnormal gray matter) in 2018. Most cases of schizencephaly are non‐familial and sporadic.^6^ The frequency is about 1.5 per 1,000,000 live births, and the frequency of epilepsy is 1 per 1650.^6^, ^7^ There is no clear gender distinction but males are slightly more dominant.^8^

The etiology is not well understood but those that cause destruction of brain tissue (eg, vascular insult; ischemia; stroke; autoimmune vasculitis; infections caused by TORCH syndrome, toxoplasmosis, syphilis, varicella‐zoster, parvovirus B19, rubella, cytomegalovirus, herpes; early prenatal injuries related to drug abuse or abdominal trauma of the mother; the frequent coincidence of other central nervous system malformations such as polymicrogyria and gray matter heterotopia; factors of inhibiting brain development); and disorders of nerve cell migration (eg, disturbed migration of primitive neuroblasts; mutations of Lhx2 or EMX2 gene) lead to schizencephaly.^5^, ^7^

Clinical severity depends on defect type, cleft size, cleft location, unilateral versus bilateral defect, the extent of the cerebral tissue damage, and the extent of anatomic abnormalities of other neural structures involved. Clinical manifestations of schizencephaly are variable and heterogeneous, such as neuro‐psychologic disorder, neurobehavioral abnormality, motor impairment, psychomotor developmental delay, intellectual dysfunction, language behavior disorder, seizure, epilepsy, microcephaly, muscle hypotonia, focal neurologic signs, spastic diparesis, hemiplegia, etc.^1^, ^2^, ^5^

Image methods give a demonstration of anatomic changes as well as in identifying other associated lesions. They delineate gray matter lining the defect and the communication with the ventricle. MRI is more sensitive than CT in detecting the clefts as well as additional abnormalities such as polymicrogyria, pachygyria, heterotopic gray matter, optic nerve hypoplasia, septo‐optic dysplasia, agenesis of the septum pellucidum and corpus callosum, etc.^8^, ^9^

Differential diagnosis of schizencephaly includes gray matter abnormalities (focal cortical dysplasia, polymicrogyria‐pachygyria, and band of heterotopic gray matter) and CSF‐containing defects (arachnoid cyst, porencephaly, ventriculomegaly, monoventricle in holoprosencephaly, hydranencephaly, hydrocephalus, agenesis of the corpus callosum with an interhemispheric cyst).^9^ Currently, there are no specific therapeutic options for schizencephaly yet. Conservative treatment is applicable in most cases. Surgical implantation of a shunt system is only applied in cases of acute life‐threatening intracranial hypertension.^1^, ^2^, ^10^ Accurate detection and diagnosis can assist in the follow‐up of schizencephaly. Gene and stem cell therapy needs more research.

Head trauma in patients with schizencephaly may be more severe than in normal patients because of poor behavioral control. Care of epidural hematoma in patients with schizencephaly should be with an interdisciplinary approach. An interprofessional team is established in this situation including a pediatrician, psychiatrist, neurologist, neurosurgeon, nurse, and pharmacist. In these patients, conservative management should be attempted; surgical treatment is only performed in case the epidural hematoma causing severe symptoms.^3^, ^5^, ^6^, ^10^

## CONFLICT OF INTEREST

The authors declare no conflicts of interest.

## AUTHOR CONTRIBUTIONS

VTH involved in writing and conceptualization. THH involved in data curation and diagnosis confirmation. VC and DTD involved in review and revision. All authors read and approved the final manuscript.

## ETHICAL APPROVAL

Ethics approval was not required for this study.

## CONSENT

Written informed consent was obtained for use of the clinical images.

## Data Availability

None.
